# Visual detection of anti‐icing fluids freezing by a low‐temperature viscosity‐sensitive aggregation‐induced emission probe

**DOI:** 10.1002/smo.20240014

**Published:** 2024-08-27

**Authors:** Honghong Zhang, Fanghui Li, Jiahong Yu, Weijun Zhao

**Affiliations:** ^1^ School of Chemistry and Molecular Engineering Key Laboratory for Advanced Materials and Joint International Research Laboratory of Precision Chemistry and Molecular Engineering Feringa Nobel Prize Scientist Joint Research Center Shanghai Key Laboratory of Functional Materials Chemistry Institute of Fine Chemicals Frontiers Science Center for Materiobiology and Dynamic Chemistry East China University of Science & Technology Shanghai China

**Keywords:** aggregation‐induced emission, anti‐icing fluids, fluorescent probe, icing detection, viscosity‐sensitive probe

## Abstract

Icing detection is critically important for preventing safety accidents and economic losses, especially concerning ice formation from invalidated anti‐icing fluids (water and ethylene glycol) under extreme conditions. Traditional technologies like ultrasonics and capacitor‐antenna face challenges with limited detection areas, lower accuracy, and susceptibility to electromagnetic interference. Here, we introduce a novel viscosity‐ultrasensitive fluorescent probe 4′,4‴‐(2,2‐diphenylethene‐1,1‐diyl) bis‐(3,5‐dicarboxylate) (**TPE‐2B4C**) based on AIEgens for monitoring ice formation of anti‐icing fluids in low‐temperature environments. **TPE‐2B4C**, consisting of four sodium carboxylate groups and multiple freely rotating benzene rings, demonstrates outstanding solubility in anti‐icing fluids and exhibits no fluorescent background signal even at low temperatures (<−20°C). Upon freezing, **TPE‐2B4C** relocates from the water phase to higher viscosity ethylene glycol, causing restriction of benzene rings and a significantly increased green fluorescence signal. **TPE‐2B4C** can successfully determine whether the anti‐icing fluids are icing from −5 to −20°C with a high contrast ratio. Due to its simple setup, fast operation, and broad applicability, our new method is anticipated to be employed for rapid, real‐time, and large‐scale icing detection.

## Graphical abstract

In this study, we designed a low‐temperature viscosity‐sensitive AIE probe called TPE‐2B4C for visual monitoring ice formation in anti‐icing liquids. This probe can dissolve in anti‐icing fluids and exhibit no fluorescent background signal even at low temperatures. After freezing, TPE‐2B4C molecules move from the water phase to higher viscosity ethylene glycol, leading to an obvious increase in green fluorescence signal.

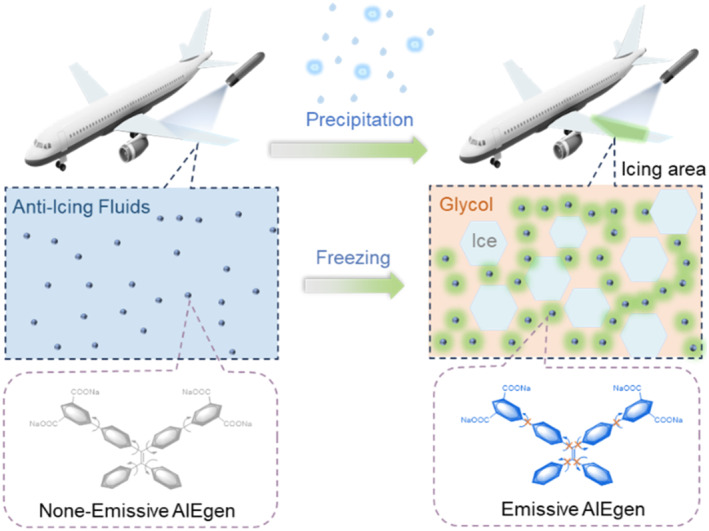

## 1 INTRODUCTION

In extreme conditions, icing poses significant hazards to power transportation,^[1]^ wind turbine,^[2]^ roads and bridges,^[3]^ and the aerospace industry.[^4^, ^5^] Particularly in aviation, aircraft icing can compromise flight performance and pose a severe threat to safety. Despite the application of anti‐icing fluids (typically a mixture of ethylene glycol and water) for protection, precipitation will dilute the anti‐icing fluids, causing the coating to freeze.^[6]^ Therefore, under icy weather conditions, it is essential to accurately detect the frozen area before takeoff for timely ice removal.^[7]^ Substantial efforts have been dedicated to determining the ice formation. Conventionally, ice formation can be directly measured by ice sensors, where electromagnetic and optical parameters are reflected as a change in electrical signals.[^8^, ^9^, ^10^] In spite of the fact that these structurally complex icing sensors have been widely used for icing detection, they suffer from poor real‐time detection reliability and small detection areas.[^11^, ^12^, ^13^]

Therefore, the development of new methods for real‐time, full‐field, and on‐site icing monitoring is critically important and is appealing for both academic research and industrial applications. Fluorescence‐based visualization detection methods have the advantages of high sensitivity,[^14^, ^15^, ^16^, ^17^, ^18^, ^19^] simplicity,[^20^, ^21^, ^22^, ^23^, ^24^] and full‐field detection[^25^, ^26^, ^27^, ^28^] compared with conventional ice sensors. However, traditional fluorophores exhibit consistent luminescence effects in both solution and frozen states, making it difficult to detect icing.^[29]^ In recent years, temperature‐responsive fluorescent probes based on the aggregation‐induced emission (AIE) mechanism have been reported to be applied for icing detection. For example, AIE fluorophores with turn‐on fluorescence upon freezing have been used to detect the freezing of aqueous and octane.[^29^, ^30^] A betaine‐based AIE antifreeze hydrogels have been fabricated, realizing the sensitive response to icing.^[31]^ Besides, a novel phosphorescent material for icing detection of water has been constructed.^[32]^ However, these probes are greatly limited to aqueous solutions or other single component solvents. It is worth noting that the vast majority of systems in practical applications are more complex than single systems. For anti‐icing fluids, which are mainly composed of double solvent with similar properties (water and ethylene glycol), there is no related research about the use of AIEgens to detect their liquid‐ice transition. Therefore, there is an urgent need to design an innovative probe to achieve high‐precision, large‐scale, and real‐time detection of anti‐icing fluid icing.

**FIGURE 1:**
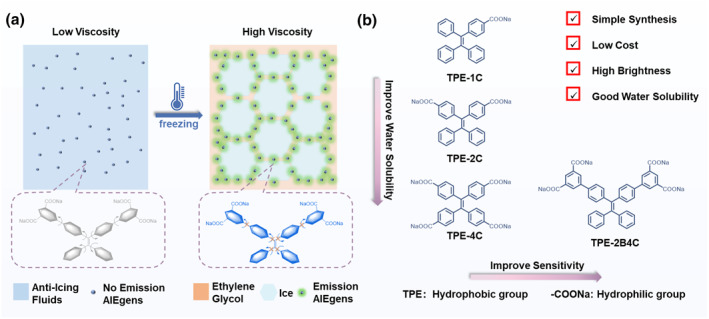
(a) Illustration of the mechanism to achieve visual detection of ice formation in ethylene glycol anti‐icing fluids. (b) Molecular structure and design of the fluorescent probe.

In this context, we designed and synthesized a viscosity‐ultrasensitive probe 4′,4‴‐(2,2‐diphenylethene‐1,1‐diyl) bis([1,1′‐biphenyl]‐3,5‐dicarboxylate) (**TPE‐2B4C**) based on AIEgens for visual monitoring ice formation in anti‐icing fluids in low‐temperature environments (Figure 1a). Probe **TPE‐2B4C** comprises two crucial elements: four water‐soluble sodium carboxylate groups, and a luminescent viscosity‐responsive tetraphenylene (**TPE**) core with additional benzene rings (Figure 1b). Adding **TPE‐2B4C** to anti‐icing fluids exhibited no fluorescent background signal even at low temperatures. When anti‐icing fluids freeze, **TPE‐2B4C** transfers from the water phase to higher viscosity ethylene glycol. This transition restricts intramolecular motion, leading to intense fluorescence emission. By utilizing **TPE‐2B4C**, we have developed an icing detection method with simple setup, fast operation, and wide applicability, enabling real‐time visualization of icing simulation on aircraft models.

## 2 RESULTS AND DISCUSSION

**FIGURE 2:**
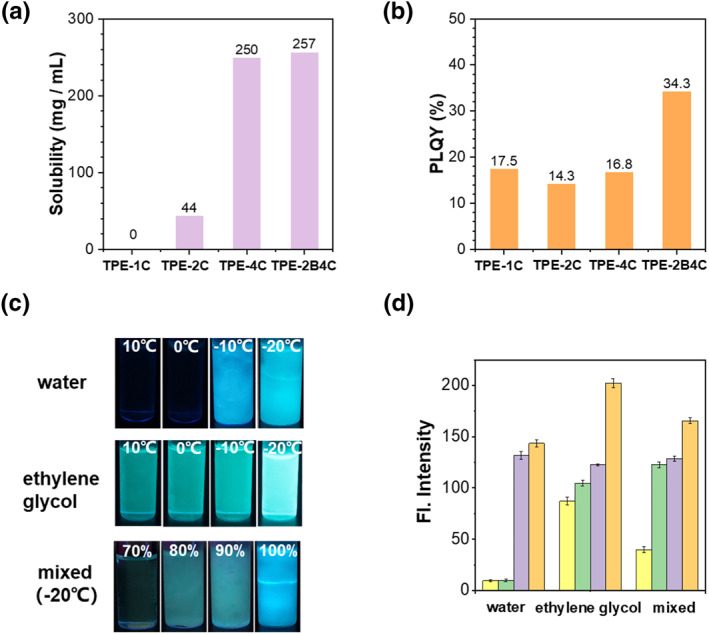
(a) The water solubility of probe TPE‐1C, TPE‐2C, TPE‐4C, TPE‐2B4C. (b) Quantum yields of TPE‐1C, TPE‐2C, TPE‐4C, TPE‐2B4C. (c) Fluorescence images of TPE‐2B4C in water and glycol at different temperatures, and in anti‐icing fluids at −20°C under the irradiation with 365 nm UV light. (d) Fluorescence intensity of TPE‐2B4C in water and glycol at different temperatures, and in anti‐icing fluids at −20°C. Statistical average value of fluorescence intensity of the selected area was obtained.

Since **TPE** is a typical AIE building block with simple synthesis and excellent AIE performance,[^33^, ^34^, ^35^] we designed a series of AIEgens by adjusting the number of substituted sodium carboxylates and benzene ring rotors in **TPE** unit (Figure 1b). The synthesis of **TPE‐2B4C** was performed via Suzuki coupling and carboxylate ester hydrolysis with a yield of up to 85% (Scheme 3). Their chemical structures were fully characterized by ^1^H NMR and ^13^C NMR. As expected, **TPE‐1C** was completely insoluble and existed as aggregates in water and anti‐icing fluids (Figure S1). As the amount of sodium carboxylate increased, the molecular water solubility gradually enhanced. The water solubility of **TPE‐4C** and **TPE‐2B4C** was 6 times higher than that of **TPE‐2C** (Figure 2a). Due to the **TPE** moiety, intensive cyan emission can be observed in poor solvent systems (acetonitrile) (Figure S2). It is noteworthy that the improved solubility ensures its application in icing detection. Furthermore, the probe's high fluorescence quantum yield is another crucial factor for sensitive detection. After adding two more benzene ring rotors, the original intramolecular charge transfer (ICT) effect from the sodium carboxylate group was inhibited,[^36^, ^37^] so fluorescence intensity of **TPE‐2B4C** was enhanced. Thus, the absolute solid‐state fluorescence quantum yield of the compound was significantly improved from 17.5% of **TPE‐4C** to 32.3% of **TPE‐2B4C** (Figure 2b).

First, we carefully explored the fluorescence performance of probes in water and ethylene glycol at different temperatures. As illustrated in Figure 2c, **TPE‐2B4C** has almost no fluorescence emission in pure aqueous solutions. With the decrease in temperature, the aqueous solution began to freeze and emitted bright blue fluorescence below zero degrees. After freezing, the fluorescence intensity significantly increases, which is 12 times that of aqueous solution (Figure 2d). Additionally, **TPE‐2B4C** exhibited good solubility in ethylene glycol solution and represented the green fluorescence shown in Figure 2c. As the temperature decreased, the fluorescence showed a visible enhancement to the naked eye. Normally, anti‐icing fluids with a content of ethylene glycol around 30% resist low temperature at −20°C. In a mixed solution with a volume fraction of 70% water and 30% ethylene glycol at −20°C, owing to existent anti freezing effect, fluorescence of probe still weak in solution (Figure 2c). As the water fraction increases, the anti‐freezing effect gradually diminishes, leading to freezing of the mixed solution. Consequently, probe molecules relocate from the water phase to ethylene glycol, accompanied by the enhancement of fluorescence intensity (Figure 2c). For Probe **TPE‐2B4C**, the fluorescence intensity increased by approximately 3‐5 fold compared to the non‐freezing solution with a water content of 70% (Figure 2d). Probe **TPE‐2C** and **TPE‐4C** only showed about 1.5 and 2 fold enhancement after freezing (Figure S3). Therefore, **TPE‐2B4C**, with appropriate sodium carboxylate and benzene groups, exhibits the highest fluorescence enhancement from solution to icing phase, rendering it the most promising candidate for visual icing detection.

**FIGURE 3:**
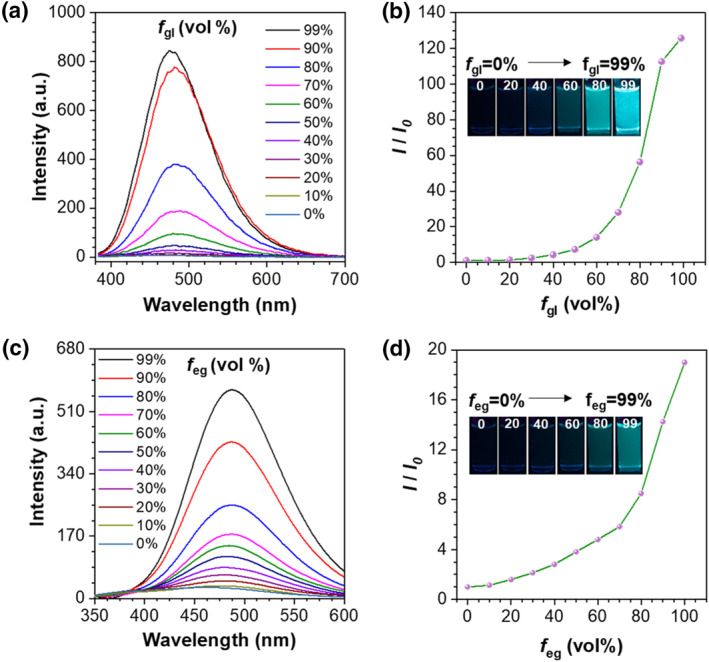
(a) Emission spectra and (b) *I/I*_0_ plots of TPE‐2B4C in a mixture of water‐glycerol with different glycerol fractions. *I* is the fluorescence intensity at 478 nm with different f_gl_ and *I*_0_ is the fluorescence intensity at *f*_gl_ = 0% (*λ*_ex_ = 365 nm). Inset: corresponding fluorescence images taken under a 365 nm UV lamp. (c) Emission spectra and (d) *I/I*_0_ plots of TPE‐2B4C in a mixture of water‐ethylene glycol system with different ethylene glycol fraction. *I* is the fluorescence intensity at 487 nm with different f_eg_ and *I*_0_ is the fluorescence intensity at *f*_eg_ = 0% (*λ*_ex_ = 365 nm). Inset: corresponding fluorescence images taken under a 365 nm UV lamp.

**FIGURE 4:**
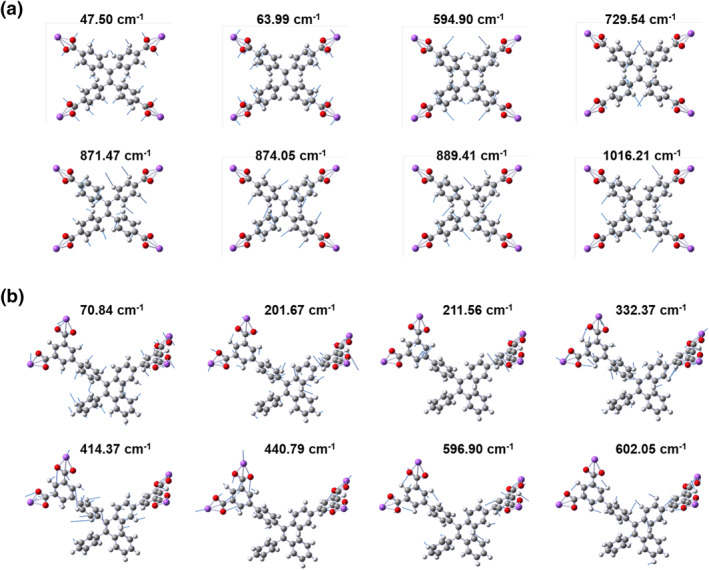
Simulated rotation vibrations of the carboxylated benzene ring of TPE‐4C (a) and (b) TPE‐2B4C.

To decipher the working mechanism of icing sensing, we investigated the fluorescent performance of designed probes in water‐glycerol systems (Figure 3a). Water‐glycerol is a typical viscosity response test system.[^38^, ^39^, ^40^] With the addition of glycerol, the viscosity of the mixture increases, thereby restricting the intramolecular rotation of molecules and significantly enhancing the luminescence efficiency. The fluorescence intensity in 99% glycerol is 126 times higher than that in pure water, indicating that **TPE‐2B4C** exhibits a highly sensitive turn‐on fluorescence response to viscosity changes (Figure 3b). Although the viscosity of ethylene glycol is significantly lower than that of glycerol, but **TPE‐2B4C** still exhibit a noticeable turn‐on fluorescence response in water‐ ethylene glycol systems (Figure 3c). In detailed, fluorescence intensity increased with the enhancement of ethylene glycol fraction from 0% to 99%, and finally resulted in a 19‐fold enhancement of fluorescence intensity at 487 nm (Figure 3d). According to AIE mechanism, we can conclude that **TPE‐2B4C** dispersed well in water‐ethylene glycol solution (working state), showing non‐emissive background signal, due to the intramolecular motion of the excited state molecules. As the water fraction increases, the water‐ethylene glycol solution freezes (failure state), leading **TPE‐2B4C** molecules to move from the aqueous phase to the higher viscosity ethylene glycol phase. Consequently, the intramolecular motion of excited state molecules is constrained, leading to a noticeable enhancement in green fluorescence. Probe **TPE‐2C** and **TPE‐4C** showed viscosity‐sensitive in water‐ethylene glycol systems but exhibited fluorescence intensity enhancement after freezing (Figure S4). To depict the rotation modes of the benzene rotors in our designed probes, density function theory calculations were performed to simulate Infrared vibrations of **TPE‐4C** and **TPE‐2B4C** (Figure 4). Except for the rotation of the benzene ring in TPE, the frequency around 211 cm^−1^, 440 cm^−1^ and 596 cm^−1^ are assigned to the rotation vibrations of additional phenyl groups of **TPE‐2B4C**. Compared to **TPE‐4C**, **TPE‐2B4C** has more vibration modes, and thus exhibit higher viscosity‐sensitivity.

**FIGURE 5:**
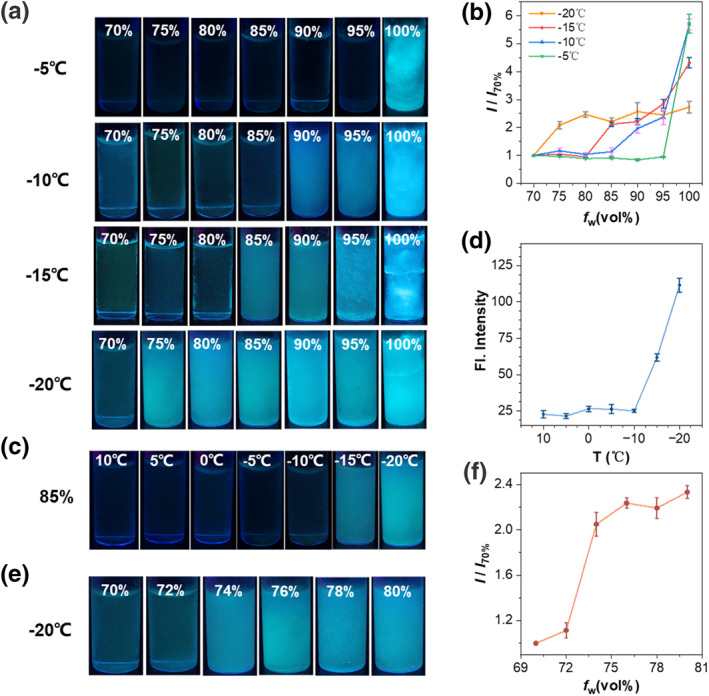
(a) Fluorescence images and (b) The contrast ratio of fluorescence intensity (*I/I*_70%_) of TPE‐2B4C in anti‐icing fluids at different temperatures. *I*_70%_ is the fluorescence intensity of 70% water by volume anti‐icing fluids and *I* is the fluorescence intensity of other water volume fraction. (c) Fluorescence images and (d) Fluorescence intensity of TPE‐2B4C in anti‐icing fluids of 85% water by volume at different temperatures. (e) Fluorescence images and (f) The ratio of fluorescence intensity (*I/I*_70%_) in anti‐icing fluids of 70%–80% water by volume at −20°C. *I*_70%_ is the fluorescence intensity of 70% water by volume anti‐icing fluids and *I* is the fluorescence intensity of other volume fraction.

Then, the working performance of probe **TPE‐2B4C** was carefully investigated at various anti‐icing temperatures and different proportions of ethylene glycol/water (Figure 5). As we all know, the anti‐icing temperature of the anti‐icing fluids increases with the increase in the volume fraction of ethylene glycol. To test this, we analyzed the fluorescence intensity change at different working temperatures (Figure 5a). At all working temperatures (from −5°C to −20°C), as the volume fraction of water increased, the anti‐icing temperature decreased, and the fluorescence intensity was significantly enhanced with a contrast ratio about 6 times (−5°C), 2–5.7 times (−10°C), 2.1–4.3 times (−15°C), 2–2.5 times (−20°C) after freezing (Figure 5b). Then, we selected 85% water by volume anti‐icing fluids and conducted more precise temperature control experiments (Figure 5c). With the decrease in temperature, the solution started to freeze at −15°C and the fluorescence increased by approximately five times at −20°C (Figure 5d). Meanwhile, we investigated a solution with a volume fraction of 70%–80% ethylene glycol, which underwent a significant transition from liquid to ice at −20°C (Figure 5e). Within a narrower range of ethylene glycol content variations, the probe detected an approximate 2.3‐fold enhancement in fluorescence intensity (Figure 5f). Therefore, the probe **TPE‐2B4C** not only facilitates the detection of low‐temperature ice over a broad range but also offers the benefits of high brightness and high contrast. With this turn‐on fluorescence signal sensing for icing, it becomes straightforward to visually ascertain whether the anti‐icing fluids have frozen.

**FIGURE 6:**
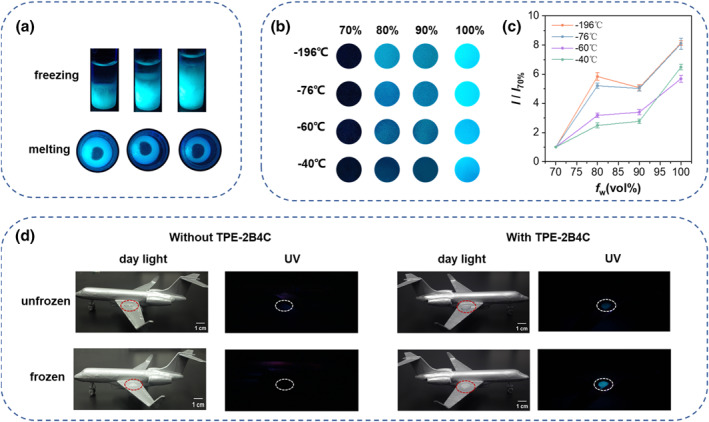
(a) Fluorescence images of TPE‐2B4C in anti‐icing fluids during freezing and melting under 365 nm UV light. (b) Fluorescence images of TPE‐2B4C in anti‐icing fluids with different freezing speeds. (c) The ratio of fluorescence intensity after freezing of TPE‐2B4C relative to 70% water by volume solution with different freezing speeds. *I*_70%_ is the fluorescence intensity of 70% water by volume anti‐icing fluids and *I* is the fluorescence intensity of other volume fractions. (d) Simulation of icing of 85% water content anti‐icing fluids on an aircraft model without (left) and with (right) at −20°C.

As shown in Figure 6a, fluorescence increases proportionally as ice grows upward from the bottom of the vial. Meanwhile, as the ice melts, the fluorescence brightness gradually decreases. The significant difference in fluorescence at the interface between the solution and ice allows for easy observation of the dynamic freezing and melting process by the naked eye. Subsequently, in order to test the performance of probe **TPE‐2B4C** under different freezing speeds, glass dishes containing anti‐icing fluids were placed in various low‐temperature environments. As shown in Figures 6a,c, it was obvious that fluorescent brightness showed a positive correlation with freezing speed. Furthermore, we applied anti‐icing fluids on the wings of the aircraft model to simulate the transition from liquid to ice in practical applications (Figures 6d,e). Notably, visually observing whether anti‐icing fluids freeze under sunlight, especially over long distances, presents challenges. After adding the probe **TPE‐2B4C**, the emergence of bright fluorescence clearly indicates the formation of icing, showcasing the potential of **TPE‐2B4C** as an icing detection sensor in practical applications of anti‐icing fluids.

## 3 CONCLUSION

In summary, we have developed a novel viscosity‐ultrasensitive fluorescent probe **TPE‐2B4C** and successfully achieved visual detection of anti‐icing fluid icing by converting morphological changes into visible fluorescence signals for the first time. Optimizing the water solubility and fluorescence responsiveness of the probe by adjusting the number of substituted sodium carboxylate and benzene rings, **TPE‐2B4C** exhibited no fluorescent background signal in solution. With the formation of ice, **TPE‐2B4C** molecules move from the water phase to higher viscosity ethylene glycol, leading to constrained benzene rings and a substantial increase in green fluorescence signal. Compared with traditional ice detection sensors, our imaging method has the advantages of simple operation and real‐time detection of a wide range, which opens up numerous opportunities for rapid and large‐scale icing detection in real applications.

## CONFLICT OF INTEREST STATEMENT

The authors declare no conflicts of interest.

## ETHICS STATEMENT

This work does not raise any ethical issues.

## Supporting information

Supporting Information S1

## Data Availability

The data that support the findings of this study are openly available in [repository name for example, “figshare”] at http://doi.org/[doi], reference number [reference number].
