# Supplementary material for: Visual detection of anti‐icing fluids freezing by a low‐temperature viscosity‐sensitive aggregation‐induced emission probe
Source: Smart Mol. 2024 Aug 27;3(1):e20240014. doi: 10.1002/smo.20240014 (PMC12117892; doi:10.1002/smo.20240014)
Supplement: Supplementary file 1 — Supporting Information S1 [file SMO2-3-e20240014-s001.docx]

Supporting Information

# Visual Detection of Anti-Icing Fluids Freezing by A Low-Temperature Viscosity-Sensitive AIE Probe

Honghong Zhang‡, Fanghui Li‡, Jiahong Yu, Weijun Zhao*

Key Laboratory for Advanced Materials and Joint International Research Laboratory of Precision Chemistry and Molecular Engineering, Feringa Nobel Prize Scientist Joint Research Center, Shanghai Key Laboratory of Functional Materials Chemistry, Institute of Fine Chemicals, Frontiers Science Center for Materiobiology and Dynamic Chemistry, School of Chemistry and Molecular Engineering, East China University of Science & Technology, Shanghai 200237

*Corresponding Authors: zhwj@ecust.edu.cn

‡ These authors contributed equally to this work

**Table of Contents**

[Experimental Procedures 2](#_Toc164705025)

[Materials and methods 2](#_Toc164705026)

[Synthesis 2](#_Toc164705027)

[Supporting Figures 7](#_Toc164705028)

[References 14](#_Toc164705029)

Experimental Procedures

Materials and methods

All reagents, solvents, starting materials, and silica gel for TLC and column chromatography were obtained from the best-known local suppliers and were used without further purification. ^1^HNMR and ^13^CNMR spectra were recorded using Bruker AM-400 Fourier transformation spectrometers operating at 400 and 101 MHz, respectively. CDCl_3_ with tetramethylsilane and D_2_O served as the solvent used as an internal reference for NMR spectra. UV-Vis absorption and fluorescence spectra were recorded on an Agilent Cary 60 spectrophotometer and Varian Cary Eclipse fluorescence spectrophotometer with a 1 cm quartz cell, respectively. The fluorescence intensity at different temperatures was analyzed by Image J software.

Synthesis

**Scheme S1** Synthetic route to TPE-1C.

**Sodium 4-(1,2,2-triphenylvinyl) benzoate (TPE-1C).** A mixture of 4-(1,2,2-triphenylvinyl) benzoic acid (100 mg, 0.27mmol), sodium hydroxide (16 mg, 0.4 mmol) and ethanol (20 mL) in a 50 mL flask was stirred at 55℃ for 12 h. The solvent was evaporated under reduced pressure to afford a white solid as product (104 mg, > 99%). TPE-1C is insoluble in common solvents.

**Scheme S2** Synthetic route to TPE-2C.

**Sodium 4,4'-(2,2-diphenylethene-1,1-diyl) dibenzoate (TPE-2C).** A mixture of 4,4'-(2,2-diphenylethene-1,1-diyl) dibenzoic acid (100 mg, 0.24mmol), sodium hydroxide (29 mg, 0.72mmol) and ethanol (20 mL) in a 50 mL flask was stirred at 55℃ for 12 h. The solvent was evaporated under reduced pressure to afford a white solid as product (107 mg, > 99%). ^1^H NMR (400 MHz, D_2_O, ppm) δ 7.60 (d, J = 8.0 Hz, 4H), 7.20-7.10 (m, 14H). ^13^C NMR (101 MHz, D_2_O, ppm) δ 175.16, 146.24, 143.28, 142.24, 139.93, 134.11, 130.91, 130.76, 128.44, 128.29, 127.82.

**Scheme S3** Synthetic route to TPE-4C.

**Sodium 4,4',4'',4'''-(ethene-1,1,2,2-tetrayl) tetrabenzoate (TPE-4C).** Compound **1** was synthesized according to our previous report.^1^ A 250 mL two necked flask equipped with a magnetic stirrer was charged with zinc dust (5.77 g, 88.25 mmol) and 50 mL THF under nitrogen atmosphere. The mixture was cooled to 0℃, and then TiCl_4_ (4.8 mL, 44.13 mmol) was added slowly by a syringe. The mixture was refluxed for 2.5 h at 90℃ and then cooled to 0℃. 4,4'-Dibromobenzophenone (6.00 g, 17.65 mmol) in THF (50 mL) was added to the mixture. Then the mixture was refluxed until TLC showing complete conversion. The reaction was quenched with saturated aqueous NH_4_Cl solution, and extracted with dichloromethane (3×80 mL). The organic layer was desiccated with anhydrous sodium sulfate for two hours, and then filtered. The solvent was removed by evaporation. The crude product is directly processed to the next step without purification.

Synthesis of Compound **2**. Compound **1** (4.00 g, 6.17 mmol) and CuCN (3.30 g, 23.8 mmol) in dry DMF (60 mL) was stirred at 150℃ for 48 h under N_2_. A mixture of ammonia (10 mL) and water (30 mL) was added to the reaction mixture and stirred at 60℃ for 2 h and then cooled to room temperature. The product was extracted with dichloromethane (3×50 mL) and the organic layer dried over sodium sulfate, filtered, and evaporated. The crude product was purified by column chromatography with dichloromethane/petroleum (1:1) as eluent to afford a white solid. Yield: 410 mg (15.4%). ^1^H NMR (400 MHz, CDCl_3_, ppm) δ 7.42 (d, J = 8.4 Hz, 4H), 7.01 (d, J = 8.4 Hz, 4H). ^13^C NMR (101 MHz, CDCl_3_, ppm) δ 141.49, 139.62, 132.80, 132.73, 131.38, 131.26, 121.30.

Synthesis of Compound **3**. Tetrakis (4-cyanophenyl) ethylene (410 mg, 0.95 mmol) and KOH (664 mg, 11.85 mmol) were refluxed for 3 days in ethylene glycol (30 mL). After cooling to room temperature, distilled water (30 mL) was added to the mixture. Subsequent washing with dichloromethane (3×30 mL) removed unreacted tetrakis (4-cyanophenyl) ethylene. The aqueous/ethylene glycol fraction was acidified using hydrochloric acid (1 mol/L) and the pale yellow precipitate was washed with water and dichloromethane. Dry the precipitant in a vacuum oven for six hours. Yield: 300 mg. A mixture of carboxylic acid (300 mg, 0.59 mmol), sodium hydroxide (95 mg, 2.36 mmol) and ethanol (20 mL) in a 50 mL flask was stirred at 55℃ for 6 h. Then the obtained precipitate is washed with ethanol and dried. ^1^H NMR (400 MHz, D_2_O, ppm) δ 7.60 (d, J = 8.4 Hz, 4H), 7.20-7.13 (m, 4H). ^13^C NMR (101 MHz, D_2_O, ppm) δ 175.21, 145.89, 141.24, 134.39, 130.92, 128.40.

**Scheme S4** Synthetic route to TPE-2B4C.

**Sodium4',4'''-(2,2-diphenylethene-1,1-diyl) bis([1,1'-biphenyl]-3,5-dicarboxylate).** Synthesis of Compound **4**. A mixture of 3,5-bis (ethoxycarbonyl) phenyl boronic acid (488 mg, 1.83 mmol), 1,1-diphenyl-2,2-di(p-bromophenyl) ethylene (300 mg, 0.61 mmol), K_2_CO_3_ (507 mg, 3.67 mmol), and Pd[P(Ph)_3_]_4_ (15 mg) was added into a 100 mL two-necked round bottom flask under an argon atmosphere. Then tetrahydrofuran (20 mL) and of H_2_O (5 mL) were added into the flask. The mixture was then refluxed at 90℃ for 12 hours, with the reaction progress monitored by TLC. At the end of the reaction, the product was extracted with dichloromethane (3×20 mL) and the organic layer dried over sodium sulfate, filtered, and evaporated. The resulting residue was purified via silica gel column chromatography with ethyl acetate/petroleum (1:5) as eluent to obtain the white solid Yield: 401 mg (85.0%). ^1^H NMR (400 MHz, CDCl_3_, ppm) δ 8.61 (t, J = 1.4 Hz, 2H), 8.41 (d, J = 1.5 Hz, 4H), 7.45 (d, J = 8.3 Hz, 4H), 7.15 (dt, J = 9.5, 7.8 Hz, 10H), 7.09 (dd, J = 6.7, 3.0 Hz, 4H), 4.43 (q, J = 7.1 Hz, 8H), 1.43 (t, J = 7.1 Hz, 12H). ^13^C NMR (101 MHz, CDCl_3_, ppm) δ 165.88, 143.67, 143.46, 142.10, 141.34, 139.46, 137.04, 131.98, 131.40, 129.13, 127.92, 126.58, 126.43, 61.47, 14.40.

The tetraester (200 mg, 0.26 mmol) and sodium hydroxide (62 mg, 1.55mmol) dissolved in 20 mL THF and 5 mL water then refluxed at 90℃ for 12 hours. The organic solvent was evaporated under vacuum. Excess sodium hydroxide is washed multiple times with ethanol to remove. ^1^H NMR (400 MHz, D_2_O, ppm) δ 8.21 (t, J = 1.5 Hz, 2H), 8.17 (d, J = 1.5 Hz, 4H), 7.53 (d, J = 8.4 Hz, 4H), 7.22 (d, J = 8.3 Hz, 4H), 7.19–7.10 (m, 10H). ^13^C NMR (101 MHz, D_2_O, ppm) δ 174.69, 143.47, 143.05, 141.30, 139.56, 137.42, 136.89, 131.99, 130.89, 129.40, 127.91, 126.26.

Supporting Figures


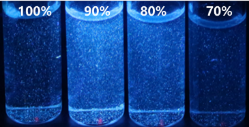


**Figure. S1** Fluorescent images of TPE-1C in water and anti-icing fluids.


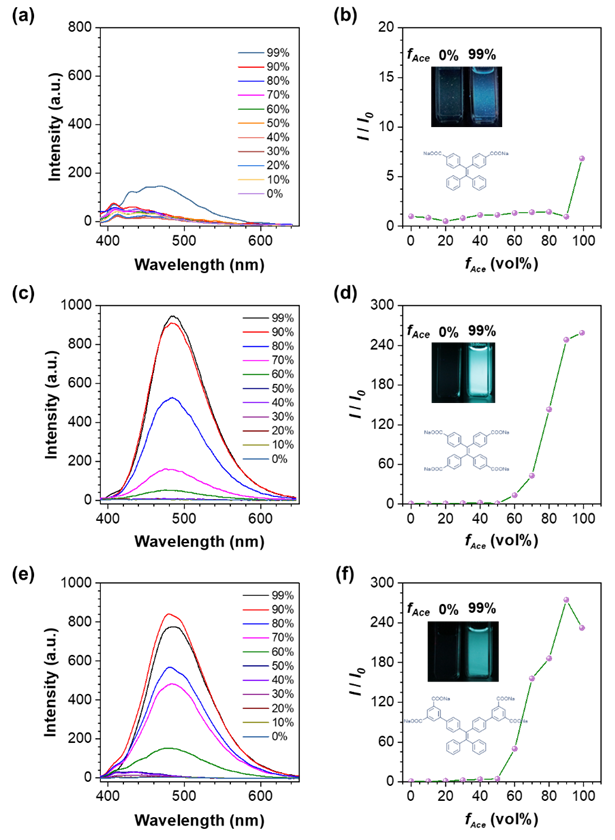


**Figure. S2** Fluorescence spectra of TPE-2C, TPE-4C, or TPE-2B4C in water / acetonitrile mixtures with different acetonitrile fractions


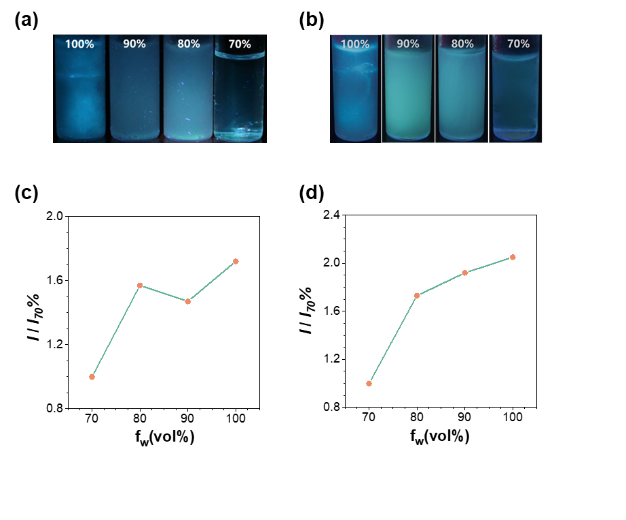


**Figure. S3** Fluorescent images of (a) TPE-1C and (b) TPE-4C in anti-icing fluids at -20℃. The contrast ratio of fluorescence intensity (*I/I_70%_*) of TPE-2B4C in anti-icing fluids at different temperatures. *I_70%_* is the fluorescence intensity of 70% water by volume anti-icing fluids and *I* is the fluorescence intensity of other water volume fraction.


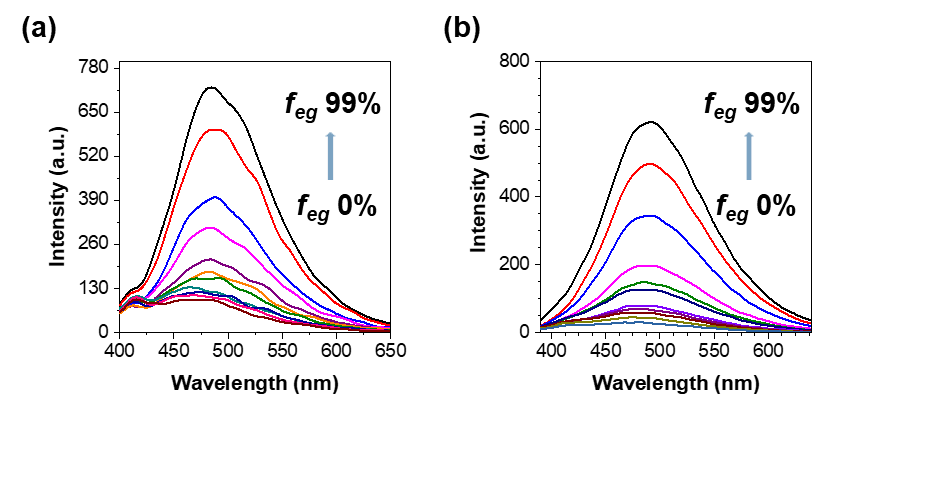


**Figure. S4** Emission spectra of (a) TPE-2C and (b) TPE-4C in a mixture of water-glycerol system (λ_ex_ = 365 nm).

**
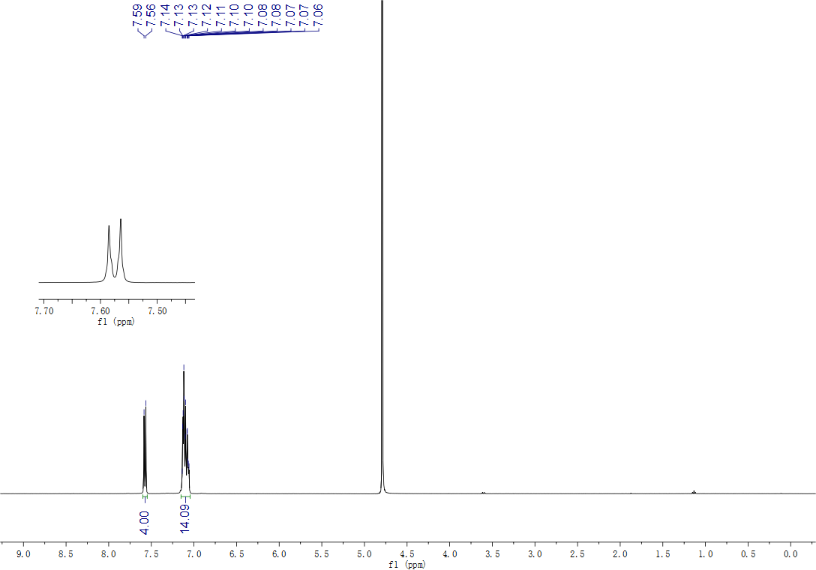
**

**Figure. S5** ^1^H NMR spectrum of TPE-2C in D_2_O.

**
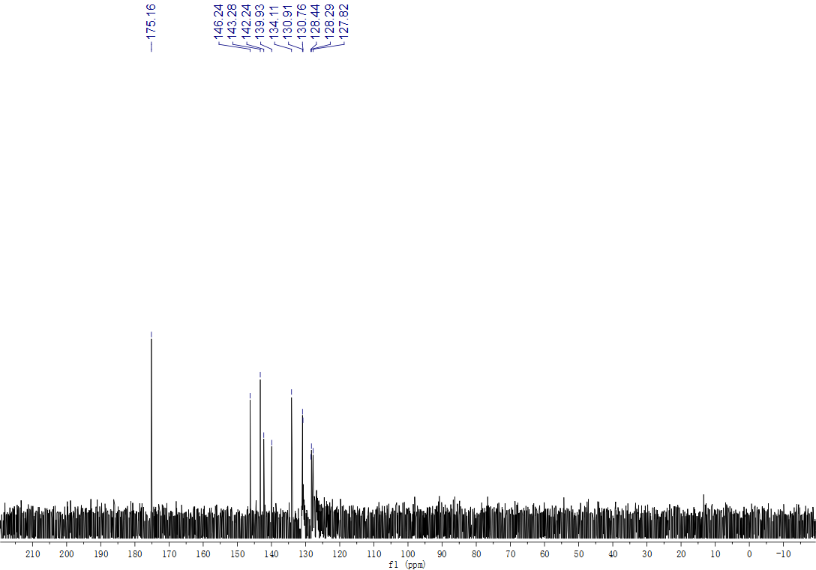
**

**Figure. S6** ^13^C NMR spectrum of TPE-2C in D_2_O.


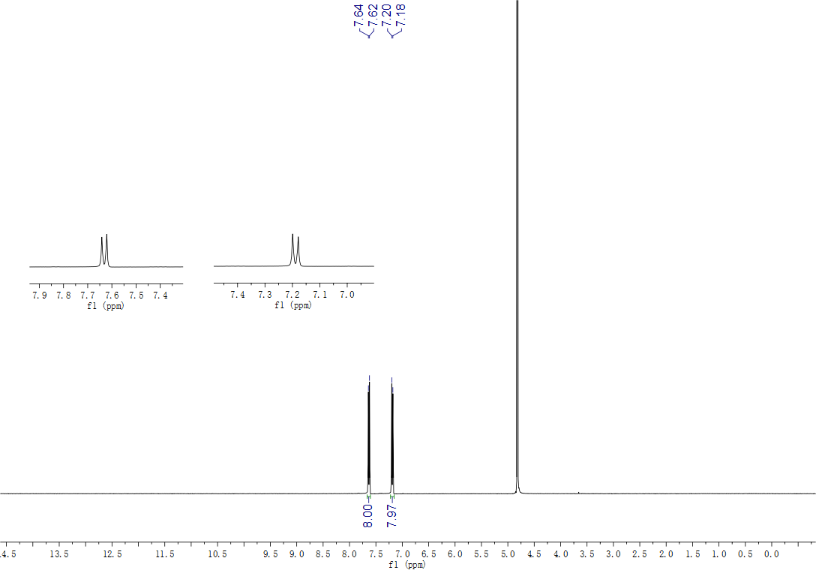


**Figure. S7** ^1^H NMR spectrum of TPE-4C in D_2_O.


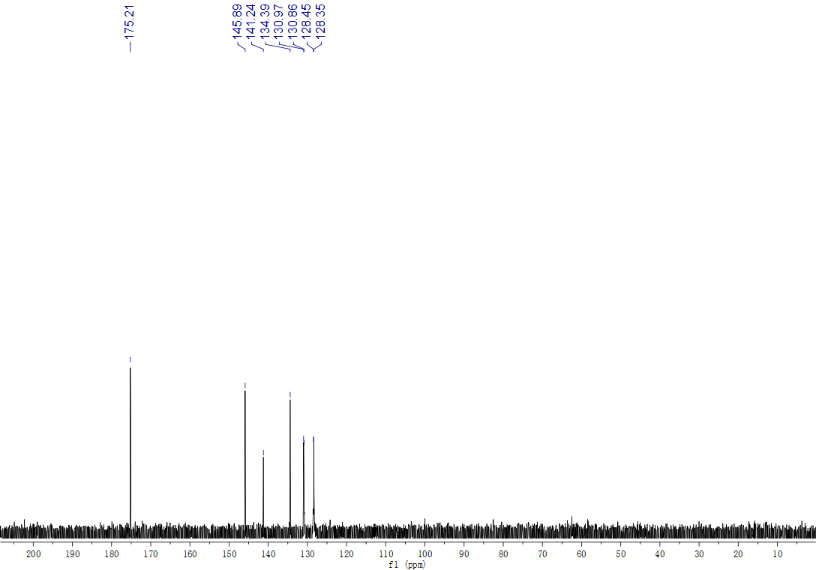


**Figure. S8** ^13^C NMR spectrum of TPE-4C in D_2_O.


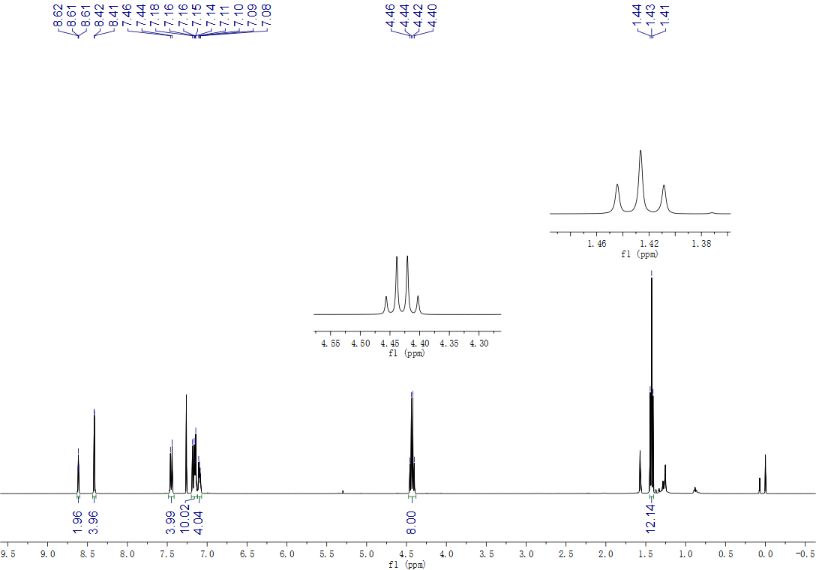


**Figure. S9** ^1^H NMR spectrum of Compound 4 in CDCl_3_.


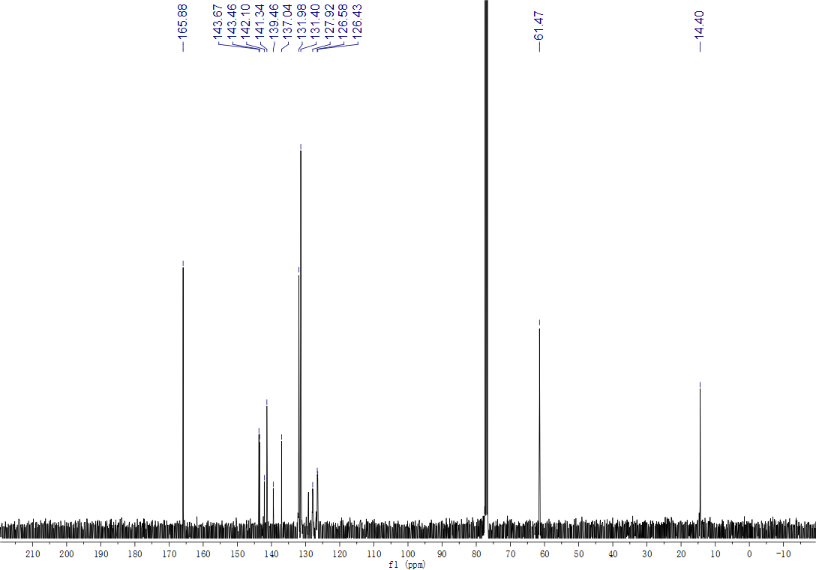


**Figure. S10** ^13^C NMR spectrum of Compound 4 in CDCl_3_.


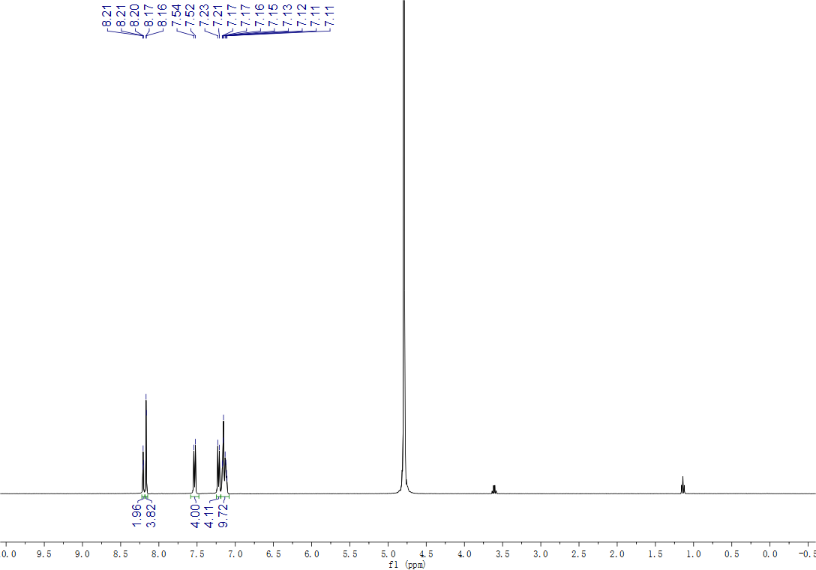


**Figure. S11** ^1^H NMR spectrum of TPE-2B4C in D_2_O.


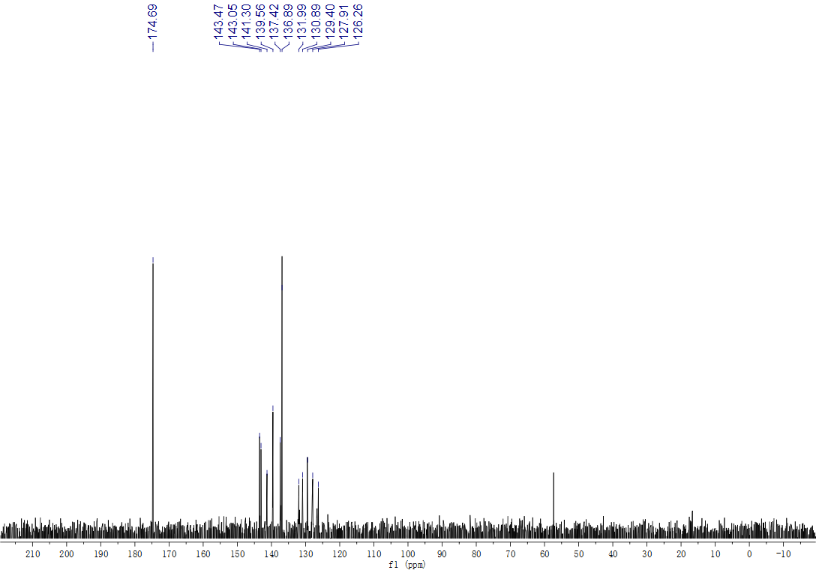


**Figure. S12** ^13^CNMR spectrum of TPE-2B4C in D_2_O.

References

1. J. Xue, W. Bai, H. Duan, J. Nie, B. Du, J. Sun, and B. Tang, *Macromolecules*, **2018**, *51*, 515762.
